# Travis County Medical Society

**Published:** 1889-01

**Authors:** B. F. Church

**Affiliations:** Secretary


### TRAVIS COUNTY MEDICAL SOCIETY.

Regular meeting held in Austin, November 1, 1888, Vice-President Justin Duffau, M. D., in the chair.

Dr. T. J. Tyner read a paper entitled "The Appearance and Behavior of the Pupil in Health and Disease."

The paper was well received by the Society without discussion.

Dr. Q. C. Smith remarked that the general practitioner should consult the specialist more frequently for aid in diagnosis.

Dr. Swearingen reported a typical case of laryngitis with fibrinous exudation, which proved fatal in forty-eight hours after the appearance of the croupal cough.

Dr. J. W. McLaughlin related a case of diphtheria. A girl aged 10 years. Had been at school during the day, complained of sore throat late in the afternoon, and during the evening her voice was somewhat hoarse. Laryngismus stridulus came on suddenly during the night, when he was called. The ordinary remedies for this condition were ordered, and he did not see her again until the next morning, then found all of the symptoms aggravated, enlarged submaxillary glands, marked dyspnea, and discovered an exudation low down in the larynx which he diagnosed as diphtheritic. One tenth of a grain of bichloride of mercury every two hours was ordered for the patient. No untoward effects, as gastritis, etc., resulted from these large doses, but improvement in all of the symptoms almost immediately followed, and recovery soon took place.

He thinks better results follow this treatment, (as recommended by Jacobi,) than any other, especially if pushed boldly in the early stages, and where asthenia is not salient.

Dr. Mathews, visiting from the city, related the cases of two children of the same family who had diphtheria, where the mouth and fauces were covered with large patches of membrane and emitted a very disagreeable odor. Both proved fatal. Another case with all symptoms of the disease, though without such a perceptible odor, which recovered. He thought this condition, in any case of diphtheria, was of serious import.

A general discussion followed as to the differential diagnosis of diphtheria and follicular pharyngitis. The Society then adjourned.

B. F. CHURCH, M. D., Secretary.

